# Novel Jumbo Biopsy Forceps for Surveillance of Inflammatory Bowel Disease: A Comparative Retrospective Assessment

**DOI:** 10.1155/2011/671659

**Published:** 2011-10-09

**Authors:** Kenneth Song, Daniel Toweill, Stephen J. Rulyak, Scott D. Lee

**Affiliations:** ^1^Division of Gastroenterology, Department of Medicine, University of Washington, Seattle, WA 98109, USA; ^2^Department of Pathology, University of Washington, Seattle, WA 98109, USA

## Abstract

*Background and Study Aims*. Most available jumbo cup forceps require a 3.7 mm biopsy channel, necessitating the use of standard-sized colonoscope. A newer jumbo forceps (Radial Jaw 4 Jumbo Biopsy Forceps [RJ4]) fits within a 3.2 mm biopsy channel, allowing use with a pediatric colonoscope. To assure the RJ4 did not alter biopsy adequacy, we compared the size and quality of specimens to a historical jumbo cup forceps (Radial Jaw 3 Max Capacity Biopsy Forceps, [RJ3 MC]). *Patients and Methods*. A retrospective comparative study of biopsies taken with either forceps. Biopsies were compared for diameter, depth, crush artifact, and acceptability for diagnosis. *Results*. 333 specimens were taken with RJ4 and 335 specimens with the RJ3 MC. Mean sample diameter was 4.45 mm and 4.55 mm for the RJ4 and RJ3 MC (*P* = 0.41). Mean depth of biopsies with the RJ4 was greater (*P* < 0.01). *Conclusions*. Biopsies from the RJ4 are similar in size and quality to biopsies from the RJ3 MC. The RJ4 has the advantage of fitting in a smaller biopsy channel.

## 1. Introduction

Jumbo cup biopsy forceps were developed to maximize the surface area of mucosal biopsies and thereby increase the diagnostic yield of the sample. The use of jumbo cup biopsy forceps has become standard practice for certain clinical scenarios such as dysplasia surveillance in Barrett's esophagus [1]. Patients with long-standing inflammatory bowel disease (IBD) are at increased risk of colonic dysplasia and cancer compared to the general population [2, 3], and professional societies have recommended frequent colonoscopic examination with jumbo cup forceps for the detection of dysplasia in patients with IBD involving the colon [4]. Detecting flat dysplasia in tissue that is endoscopically normal is entirely dependent on adequate sampling of the affected surface area. 

While standard clinical practice has included the use of jumbo cup forceps for IBD surveillance biopsies, this forceps requires the use of an adult colonoscope because a 3.7 mm working biopsy channel is necessary to accommodate the typical jumbo cup biopsy forceps. Pediatric colonoscopes have been increasingly used for colonoscopy in adults both as a matter of physician preference and potential for improved patient comfort [5, 6]. However, most currently available pediatric colonoscopes have a 3.2 mm working channel which is not of sufficient diameter to allow passage of the standard jumbo cup biopsy forceps. A new large diameter biopsy forceps, the Radial Jaw 4 Jumbo Biopsy Forceps (RJ4) [Boston Scientific, Inc., Boston, Mass, USA] has been developed to fit within the working channel of a pediatric colonoscope. The goal of the present study was to compare the size and the quality of biopsies taken with the new RJ4 forceps to those taken with a historical jumbo cup biopsy forceps.

## 2. Materials and Methods

### 2.1. Patients

IBD patients presenting to the University of Washington (UW) Medical Center between July 2002 and November 2006 for colonoscopy were studied retrospectively. The protocol was reviewed and approved by the local institutional review board. Patients were included if they were at least 18 years of age, carried a diagnosis of IBD (Crohn's or ulcerative colitis), and had undergone colonoscopy for dysplasia surveillance. An equal number of patient cases were selected based on use of the RJ4 or historical jumbo forceps (Radial Jaw 3 Max Capacity Biopsy Forceps, Boston Scientific, Inc., Boston, Mass, USA. [RJ3 MC]). 

Nine patients underwent colonoscopy and biopsies with the RJ4 forceps, and specimens were compared to a historical cohort of nine patients who underwent biopsies with the RJ3 MC. 

### 2.2. Biopsy Forceps

**Figure 1:**
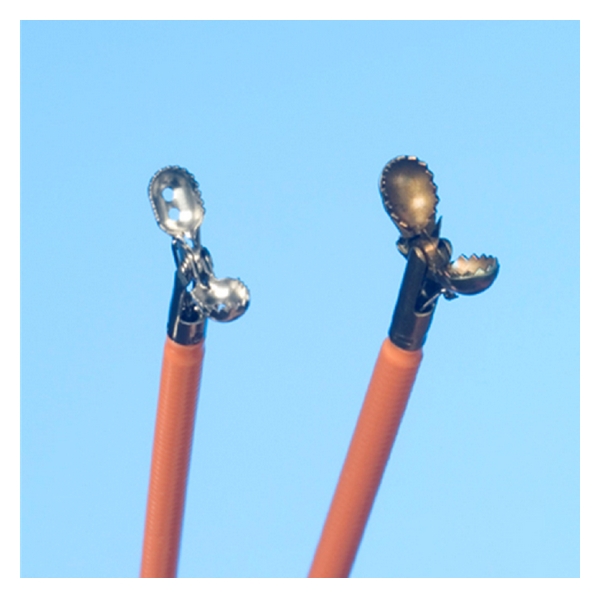
RJ4 (left) and standard large diameter forceps (right). Both are straight shaft forceps with a cup design.

 Both forceps used in the study are similar straight shaft forceps with a cup design (Figure 1). The RJ4 forceps has a shorter jaw length and smaller jaw width than the RJ3 MC forceps (jaw length: 3.8 mm versus 4.3 mm and jaw width: 2.8 mm versus 3.3 mm, for the RJ4 and RJ3 MC Biopsy Forceps, resp.). However, the maximum jaw opening of the forceps is greater for the RJ4 (8.8 mm) compared to the RJ3 MC (8.5 mm). The RJ4 forceps design also incorporates 2 fenestrated holes in the jaws which may aid in obtaining larger tissue samples. 

### 2.3. Endoscopic Procedure

A single gastroenterologist (S. D. Lee) performed all colonoscopies on each of the patients. The Olympus CF-Q160 colonoscope (Olympus America Inc., Center Valley, Pa, USA) was used with the RJ3 MC forceps and the Olympus PCF-Q160 colonoscope was used with the RJ4 forceps. The size of the working channel necessary for each of the biopsy forceps varied, and; therefore, two different endoscopes were needed to accommodate the individual forceps. While the endoscopes had different working channels; the other qualities of the endoscopes were otherwise similar. In addition, the protocol by which the biopsies were taken was identical. Dysplasia surveillance was performed using a standard protocol to maximize the detection of dysplasia, which consisted of four quadrant biopsies taken beginning in the cecum and every 10 cm throughout the colon down to 25 cm from the anal verge and then every 5 cm to the anus [7]. Each biopsy sample was obtained in a single passage under direct endoscopic visualization and was immediately placed in Hollande's fixative medium. 

### 2.4. Histologic Evaluation

Biopsy specimens were oriented by an experienced histotechnologist and embedded in paraffin. Sections (3 *μ*m to 4 *μ*m each) were mounted on glass slides and stained with hematoxylin and eosin (H&E). For each biopsy specimen, multiple levels (three to four sequential slides with six to twelve serial sections each) were examined, and the largest tissue sections were selected for subsequent measurement. For comparison, only those biopsies that were taken in the colon for dysplasia surveillance were selected. We excluded biopsies from the terminal ileum as well as biopsies of any polyps or other masses from our analysis. 

All biopsy specimens were reviewed by a single pathologist (D. Toweill), who was blinded to the type of forceps used. Each biopsy specimen was analyzed for diameter, depth, crush artifact, and acceptability for diagnosis. Metric measurements were made using a calibrated optical micrometer on an Olympus CX41 microscope (Olympus America Inc., Center Valley, Pa, USA). For diameter and depth, the longest and the widest dimensions, respectively, of the specimen were measured. Depth was also assessed based on histological depth using a 4 point scale: 1 = superficial, 2 = mucosa (at the level of the lamina propria), 3 = muscularis mucosa, 4 = submucosa. Crush artifact was assessed based on a 3 point scale: 1 = minimal, 2 = moderate, 3 = severe. Acceptability for diagnosis was assessed on a 3 point scale: 1 = inadequate, 2 = suboptimal, 3 = adequate. 

### 2.5. Statistical Analysis

The primary outcome for analysis was diameter of the biopsy specimen. Sample size calculation was conducted a priori, and we determined that 300 samples per group would be sufficient to detect a 0.2 mm difference in diameter to yield 80% power with 5% type 1 error. For continuous data, the mean standard deviation and 95% confidence intervals were calculated, and continuous data were analyzed using Student's *t-*test. Categorical data were analyzed using the chi-squared test or Fisher's exact test, as appropriate. All *P* values were two-sided, and the level of significance was *P* < 0.05.

## 3. Results

Clinical and demographic characteristics for the 18 patients are given in Table 1. All patients underwent colonoscopy for dysplasia surveillance and had a complete examination including evaluation of the terminal ileum. There were more male patients and more patients with Crohn's disease in the RJ4 forceps group, but this was not of statistical significance. All colonoscopies were complete. 

**Figure 2:**
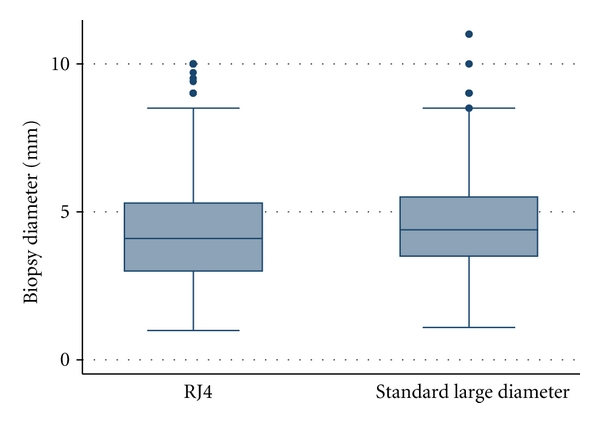
Comparison of mean biopsy diameter for specimens taken with the RJ4 forceps and standard large diameter forceps. There was no significant difference in diameter of specimens obtained with the two forceps (*P* = 0.41).

A total of 333 biopsy specimens taken with the RJ4 forceps were compared with 335 biopsy specimens taken with the RJ3 MC forceps (Table 2). There was no significant difference in mean diameter of biopsy sample obtained with the forceps (*P* = 0.41) (Figure 2). The mean diameter of biopsies obtained with the RJ4 forceps was 4.45 mm (95% CI: 4.25–4.64 mm) compared with a mean diameter of 4.55 mm (95% CI: 4.39–4.72 mm) for RJ3 MC forceps. The measured depth of biopsies taken with the RJ4 forceps (mean depth: 0.66 mm, 95% CI: 0.64–0.68 mm) was greater than the depth of biopsies taken with the RJ3 MC forceps (mean depth: 0.55 mm, 95% CI: 0.53–0.57 mm) (*P* < 0.01). However, there was no difference in the histologically determined depth of biopsies between the two forceps (*P* = 0.15). 

The quality of the biopsy specimens obtained with the two specimens was similar. The majority of specimens obtained with either forceps had only minimal crush artifact, and the degree of crush artifact did not differ significantly between the two forceps (*P* = 0.15). Although the majority of specimens obtained with either forceps were subjectively judged to be adequate for diagnosis, there were a greater proportion of “suboptimal” samples obtained with the RJ4 forceps (9.3%) compared with the RJ3 MC forceps (2.7%) (*P* < 0.01). However, very few specimens obtained with either forceps were deemed inadequate for diagnosis with either forceps (*n* = 4 for each forceps). 

No endoscopic or forceps-related complications in either of the patient groups were identified. The solo endoscopist also did not notice any difference in procedure time or technical difficulty with either forceps, although these outcomes were not quantified. 

## 4. Discussion

Detection of flat dysplasia in tissue that appears endoscopically normal is dependent on adequate sampling of the affected surface area. Without adequate sampling, there can be a significant reduction in the sensitivity to detect dysplasia, lowering the utility of such screening. The historical jumbo cup forceps were produced to improve the size of the biopsies attained, thereby, increasing the diagnostic yield of the sample. The increase in biopsy sample size had previously been accompanied by an associated increase in the size of the forceps. The historical jumbo cup forceps requires a 3.7 mm working channel on an endoscope, restricting the types of endoscopes with which they may be used. The RJ4 forceps was designed to increase the size of the biopsy sample attained, but only requiring a 3.2 mm working channel. While the RJ4 was thought to obtain similar size biopsies to historical jumbo cup forceps, at the time we conducted our study, there were no human clinical studies comparing the size of samples obtained. 

In our study, the diameter, histologic depth, and crush artifact of the biopsy samples taken with the RJ4 forceps were statistically similar to the samples obtained using the RJ3 MC. Although we found that more specimens obtained with the RJ4 forceps were graded suboptimal for diagnosis, very few specimens were inadequate for diagnosis. We did not find any association between diameter, depth, or crush artifact and the likelihood of a suboptimal specimen. We speculate that the orientation of the biopsy, fragmentation, presence of lymphoid aggregates, and lack of uniformity of depth across the entire biopsy specimen could have contributed to the suboptimal specimens. For those patients who get numerous biopsies as in the case of IBD dysplasia surveillance, we believe that the minority of suboptimal specimens amongst the total number of samples is unlikely to affect the final diagnosis, although the present study was not powered to answer this question. It is worth emphasizing that all of the suboptimal biopsies were still considered adequate for evaluation of the presence of the histologic changes of IBD and dysplasia. However, caution regarding the RJ4 forceps may be warranted in situations where very few biopsies are obtained. In the situation that numerous biopsies are not being taken and the physician consider a pediatric colonoscope critical to the procedure, we would suggest using a RJ4 MC for taking biopsies. 

A recently published study by Elmunzer et al. also evaluated the Radial Jaw 4 Jumbo Biopsy Forceps in IBD patients undergoing surveillance colonoscopy [7]. In this study, the authors determined adequacy of biopsy specimen based on diameter, crush artifact, and histologic depth of penetration. While this study is similar to our current study, the authors actually compared the RJ4 to a commonly used biopsy forceps requiring a 2.8 mm working channel, the Radial Jaw 3 Large Capacity Biopsy Forceps ([RJ3 LC], Boston Scientific, Inc.). The maximal diameter of the RJ3 LC is actually smaller than the RJ4 and only requires a 2.8 mm working channel. Using this set of criteria, they concluded the RJ4 was superior to RJ3 LC. Applying the same criteria to our biopsy samples, we find that the RJ4 has fewer adequate samples compared to the RJ3 MC forceps (63% versus 79%). It is difficult to compare the results between these two studies as they compared a different forceps to the RJ4. Given the known dimensions of the biopsy forceps used in the study by Elmunzer et al., it is not surprising that the authors found that the samples obtained with the RJ4 were larger than the samples obtained with the RJ3 LC. In contrast, our study showed no significant differences between the samples. However, it is reassuring to know that there is no significant difference between the RJ4 and RJ3 MC samples, as the physical dimensions of the RJ3 MC are larger than the RJ4 and have the added benefit of being able to fit in a pediatric colonoscope. The ability to use either an adult or pediatric colonoscope gives physicians a wider range of choices in instruments, and the pediatric colonoscope may allow for increased patient comfort and provider satisfaction with surveillance colonoscopy. 

Previous studies have suggested that maximizing the sampled epithelial surface area by taking multiple, large biopsies can enhance detection of dysplasia in IBD [8]. Therefore, maximizing diameter could be argued as the most important factor when obtaining a biopsy specimen. Crush artifact is likely another important factor, but assessing depth of the biopsy into layers beneath the epithelial surface may be less relevant, at least in the case of dysplasia detection. 

Limitations to our current study include the retrospective study design, performance at a single center with all procedures done by one endoscopist, and review of specimens by a single pathologist. While measurements were not performed prospectively, we believe that the retrospective measurements are clinically relevant because these represent the final product that the pathologist will review. It should also be noted that the present study included only IBD patients, although we expect our findings could be generalized to other situations where biopsies are taken during colonoscopy. The utility of the RJ4 forceps in upper endoscopy procedures, however, may require separate evaluation, although it should be noted that these forceps should not be used with upper endoscopes with a working channel smaller than 3.2 mm. 

In conclusion, our results demonstrate that for obtaining colonic surveillance biopsies in IBD patients, the RJ4 forceps is comparable to a historical jumbo cup forceps, the RJ3 MC which was designed to maximize sample size. However, given the difference in inadequate samples in the RJ4 group, until further studies can show that this is not clinically significant, gastroenterologists should consider this difference in inadequate samples in our study when choosing to use the RJ4 in surveillance biopsies and consider using the RJ3MC unless there is an anatomic reason to use a pediatric colonoscope. As all of the biopsies were performed by a single endoscopist in a relatively small number of patients, we feel that while the RJ4 shows promise in this population; before the general use of the RJ4 can be recommended, further studies including a larger number of subjects, with multiple endoscopists taking biopsies should be undertaken. Further it may be adviseable in future studies to use only adult colonoscopies and blinding the endoscopist to the biopsy force being used to reduce any bias. If such studies confirm our results, the general use of the RJ4 will allow the use of either a pediatric or an adult colonoscope without concern of affecting clinical outcomes and may allow for increased patient comfort and provider satisfaction with surveillance colonoscopy. 

## Figures and Tables

**Table 1:** Clinical and demographic characteristics of inflammatory bowel disease patients.

	RJ4 (*n* = 9)	Standard large diameter (*n* = 9)
Mean age (years)	49.2 (range: 20–90)	40.2 (range: 18–72)

Sex		
Male	7 (78%)	4 (44%)
Female	2 (22%)	5 (56%)

Diagnosis		
Crohn's	3 (33%)	1 (11%)
Ulcerative colitis	6 (67%)	8 (89%)

Extent of exam		
Terminal ileum	9 (100%)	9 (100%)

**Table 2:** Comparison of biopsy size and quality between RJ4 and standard large diameter forceps.

	RJ4 (*n* = 333)	Standard large diameter (*n* = 335)	
Diameter mean (mm)	4.45 (95% CI: 4.25–4.64)	4.55 (95% CI: 4.39–4.72)	*P* = 0.41

Depth mean (mm)	0.66 (95% CI: 0.64–0.68)	0.55 (95% CI: 0.53–0.57)	*P* < 0.01

Depth level			
Mucosa	14% (48)	10% (32)	*P* = 0.15
Muscularis mucosa	67% (224)	72% (221)	
Submucosa	18% (61)	19% (62)	

Crush artifact			
Minimal	91% (304)	94% (316)	*P* = 0.15
Mild	9% (29)	5% (18)	
Severe	0% (0)	0% (1)	

Acceptability for diagnosis			
Adequate	89% (298)	96% (316)	*P* < 0.01
Suboptimal	9% (31)	3% (9)	
Inadequate	1% (4)	1% (4)	
